# The 'help' question doesn't help when screening for major depression: external validation of the three-question screening test for primary care patients managed for physical complaints

**DOI:** 10.1186/1741-7015-9-114

**Published:** 2011-10-18

**Authors:** Patrick Lombardo, Paul Vaucher, Nader Haftgoli, Bernard Burnand, Bernard Favrat, François Verdon, Thomas Bischoff, Lilli Herzig

**Affiliations:** 1 Institute of General Medicine, University of Lausanne, Lausanne, Switzerland; 2 Clinical Epidemiology Centre, Institute of Social and Preventive Medicine, University of Lausanne, Lausanne, Switzerland; 3 Department of Ambulatory Care and Community Medicine, University of Lausanne, Lausanne, Switzerland

## Abstract

**Background:**

Major depression, although frequent in primary care, is commonly hidden behind multiple physical complaints that are often the first and only reason for patient consultation. Major depression can be screened by two validated questions that are easier to use in primary care than the full *Diagnostic and Statistical Manual of Mental Disorders*, fourth edition (DSM-IV) criteria. A third question, called the 'help' question, improves the specificity without apparently decreasing the sensitivity of this screening procedure. We validated the abbreviated screening procedure for major depression with and without the 'help' question in primary care patients managed for a physical complaint.

**Methods:**

This diagnostic accuracy study used data from the SODA (for 'SOmatisation Depression Anxiety') cohort study conducted by 24 general practitioners (GPs) in western Switzerland that included patients over 18 years of age with at least a single physical complaint at index consultation. Major depression was identified with the full Patient Health Questionnaire. GPs were asked to screen patients for major depression with the three screening questions 1 year after inclusion.

**Results:**

Of 937 patients with at least a single physical complaint, 751 were eligible 1 year after index consultation. Major depression was diagnosed in 69/724 (9.5%) patients. The sensitivity and specificity of the two-question method alone were 91.3% (95% CI 81.4 to 96.4) and 65.0% (95% CI 61.2 to 68.6), respectively. Adding the 'help' question decreased the sensitivity (59.4%; 95% CI 47.0 to 70.9) but improved the specificity (88.2%; 95% CI 85.4 to 90.5) of the three-question method.

**Conclusions:**

The use of two screening questions for major depression was associated with high sensitivity and low specificity in primary care patients presenting a physical complaint. Adding the 'help' question improved the specificity but clearly decreased the sensitivity; when using the 'help' question, four out of ten patients with depression will be missed, compared to only one out of ten with the two-question method. Therefore, the 'help' question is not useful as a screening question, but may help discussing management strategies.

## Background

Major depression is found in 3.9% of the general population in Europe [1] and a prevalence of 5% to 14% has been reported in primary care patients [2-6]. In a more recent meta-analysis the rate of depression was even of 17% to 19% [7]. However, major depression is commonly hidden behind multiple and sometimes unexplained physical complaints that are often the first and only reason for patients to request consultation [8-12]. Detecting mental disorders in the presence of such complaints is thus an important challenge for general practitioners (GPs) [13]. To help GPs detect major depression, a screening tool containing two questions has been derived from the *Diagnostic and Statistical Manual of Mental Disorders*, fourth edition (DSM-IV) criteria and validated [14]. These questions are simple, respectful, easy to integrate into the consultation, and require less time than the full DSM-IV criteria. Arroll *et al. *[15,16] suggested the addition of a third question called the 'help' question, in which the patient is asked whether they would like help regarding the issues raised by the first two screening questions. This new screening tool was reported to result in increased specificity (from 67% to 89%) not accompanied by decreased sensitivity (from 97% to 96%). In general, the addition of a mandatory qualifying question to a screening tool usually decreases the sensitivity and increases the specificity of the test, unless the added question is perfectly discriminatory.

Since most primary care patients are usually followed by their GP for many years, we conducted a novel investigation into the utility of these screening procedures over time. We examined the contribution to diagnosis of the two screening questions and the additional 'help' question in patients previously seen by a GP for a physical complaint (index consultation) and followed-up for a year. The accuracies of the two-question and three-question screening methods were explored across subgroups defined by age, gender, education level, migration status, presence of other mental disorders (anxiety, somatoform disorder, alcohol abuse), and presentation of major or minor depression at the time of index consultation.

## Methods

This diagnostic accuracy study was nested within a larger cohort study on the occurrence and correlations of depression, anxiety, and somatoform disorders (the SODA (for 'SOmatisation Depression Anxiety') cohort study [17]) in primary care patients with physical complaints who were followed over 1 year. Data were collected in western French-speaking Switzerland by 21 GPs in private practice and 3 medical doctor (MD) trainees from 1 academic primary care centre from November 2004 to March 2007. This study protocol was approved by the State Ethics Committee of the Canton of Vaud (Prot.100/04).

### Patients and follow-up

This study, conducted 1 year after the index consultation, included consenting patients aged 18 years and over who presented with at least 1 physical complaint during the index consultation at 1 of 22 recruiting centres. Patients with vital emergencies, dementia, intellectual deficiency, inability to understand French, or acute psychiatric diseases that prevented the patient from answering appropriately were excluded. The GPs included one patient per each half-day of consultation. To minimise selection bias, patients eligible for inclusion were selected by each GP using a pre-established, daily, randomised rank order list, thus defining each eligible patient for every half-day. In the academic primary care centre all eligible patients were enrolled (MD trainees see fewer patients) nevertheless more patients could not be included, mainly due to language barriers. GPs completed a case report form for each patient. Each patient received a self-administered questionnaire that was either to be completed in the waiting room or returned by mail in the next few days. Patients were followed-up by their GPs as needed according to usual practice. The 1-year follow-up consultation took place during a scheduled visit 9-15 months after the index consultation. Patients who did not consult their physicians spontaneously during the 1-year follow-up were invited by phone to plan a visit within the next 3 months. Data collected during the follow-up consultation allowed the assessment of the accuracy of the screening questions in detecting major depression.

The participating primary care physicians were all trained in family practice or general internal medicine and worked in primary care settings. These physicians were trained in the use of the three screening questions for major depression. GPs were allowed to investigate depression only after they asked the three screening questions. Physicians were blinded to the reference standard results of both the initial and follow-up consultations, but were not necessarily blinded to the patient's depression status.

### Questionnaires

During the index and follow-up consultations, GPs read out the two screening questions for major depression: 'During the past month have you often been bothered by feeling down, depressed, or hopeless?' and 'During the past month have you often been bothered by little interest or pleasure in doing things?'. Patients responding positively to either of these questions were asked the 'help' question: 'Is this something with which you would like help?' with three possible responses: 'no', 'yes, but not today', or 'yes'. These three screening questions were translated from English to French and then reverse translated. Patients responding positively to either of the first two questions were considered 'positive' for the two screening questions. Patients who responded positively to either of the two questions and to the 'help' question ('yes' or 'yes, but not today') were considered 'positive' for the three screening questions. All other patients were considered 'negative'.

After the consultation, the patients independently completed the reference standard questionnaire (full Patient Health Questionnaire (PHQ)) [3,18,19], a validated French version of the self-reported Primary Care Evaluation of Mental Disorders (PRIME-MD) [20] questionnaire. This questionnaire was designed to detect mental disorders in primary care practice, including depression, anxiety, alcohol abuse, and eating and somatoform disorders. To classify whether patients had major depression, we used nine questions corresponding to DSM-IV criteria (questions 2a to 2i) [18]. Patients who responded positively to at least one of the first two screening questions and to five or more of the nine questions were considered to have major depressive syndrome. Minor depression was considered present when three or four of the nine questions were answered positively and at least one of the two core questions.

Anxiety, somatoform disorder, alcohol abuse and exposure to psychosocial stressors were assessed with PHQ questions. Patients were considered to be exposed to a psychosocial stressor if they reported being bothered a lot by at least one of the ten stressors assessed with question 12 of the full PHQ [18] (1, health; 2, weight or appearance; 3, having little or no sexual desire or pleasure during sex; 4, difficulties with husband/wife, partner/lover or boyfriend/girlfriend; 5, the stress of taking care of children, parents or other family members; 6, stress at work or outside of the home or at school; 7, financial problems or worries; 8, having no one to turn to when having a problem; 9, something bad that happened recently; 10, thinking or dreaming about something terrible that happened in the past). Sociodemographic questions included age, gender, and nationality (dichotomised into Swiss or non-Swiss). Professional education included eight categories summarised in a dichotomised variable: presence or absence of fully achieved training beyond compulsory school.

Questionnaires were sent to the data centre, and all variables were double entered and checked. A researcher, blinded to index consultation results, determined which patients presented PHQ criteria for major depression.

### Statistical methods

The sample size necessary to obtain a 10%-wide interval around a 70% expected sensitivity (α = 0.05) was calculated, assuming a 10% prevalence of major depression. The expectation of 20% loss to follow-up led to a total of 947 patients required for inclusion, a figure that was rounded to 1,000 patients.

Sensitivity, specificity, positive and negative likelihoods, and predictive values were calculated, with their respective 95% confidence intervals (95% CIs), to determine screening test accuracy. Sensitivity, specificity, and 95% CIs were also calculated for subpopulations stratified by age, gender, nationality, education level, anxiety, somatoform disorder, depression status at the index consultation, and exposure to a psychosocial stressor. Although these variables were predefined before analysis, this study was not sufficiently powerful to detect significant clinical differences between subgroups. The effects of these factors on the screening method were estimated by likelihood ratio test comparing logistic regression models with or without an interaction term. Characteristics of the patients (age, gender, level of education, and depression at index consultation) were compared between patients included and those excluded from the analysis to assess potential selection bias.

## Results

**Figure 1:**
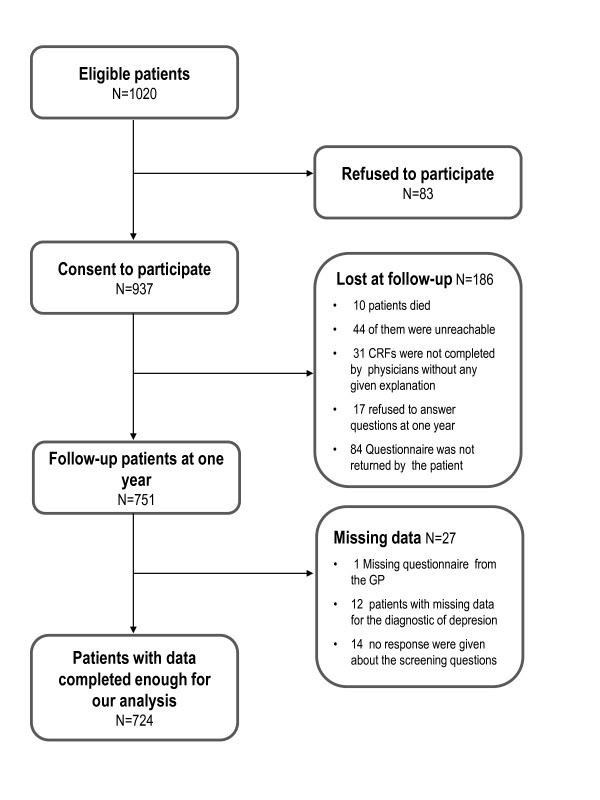
**Flowchart of eligible patients**.

Between November 2004 and July 2005, 937 patients were included in the present study. At 1 year after inclusion, 751 patients agreed to be questioned (Figure 1). A total of 12 patients did not answer all PHQ questions, making it impossible to know whether they were suffering from depression, and the physician did not report the results of 3 screening questions for 15 other patients. Thus, 724 patients were included in the analysis. The included patients were similar to those excluded regarding gender (63.3% of women in the group included vs 62.4%), age of 65 years or over (29.8% vs 25.3%), education level (79.9% vs 79.8%), and presence of major depression at the index consultation (11.3% vs 14.0%). Most patients (91.3%) were recruited from private practices, with the number of patients from each practice ranging from 6 to 58. Patients were mainly women (63.3%) and had a mean age of 54.7 years (SD 17 years). The most frequent diagnoses for the main physical complaint were musculoskeletal (29.9%) or digestive (8.4%). In 94 patients (13%), a mental disorder was considered to be related to the initial physical complaint. During the year of follow-up, 83.1% of patients visited their GP at least once, and 40.4% received psychotherapeutic care from their GP. Psychotropic drugs were used by 34.2% of the patients and 8.1% were referred to either a psychiatrist or a psychologist. At 1 year after the index consultation the prevalence of major depression was of 9.5%.

The depression screening test administered by GPs was completed on the same day as the reference test (PHQ) by 59.3% and within 1 week by additional 25% of patients. Physicians did not report any adverse effects of using the three screening questions. GPs did not report an answer to the 'help' question in five patients (0.7%).

**Figure 2:**
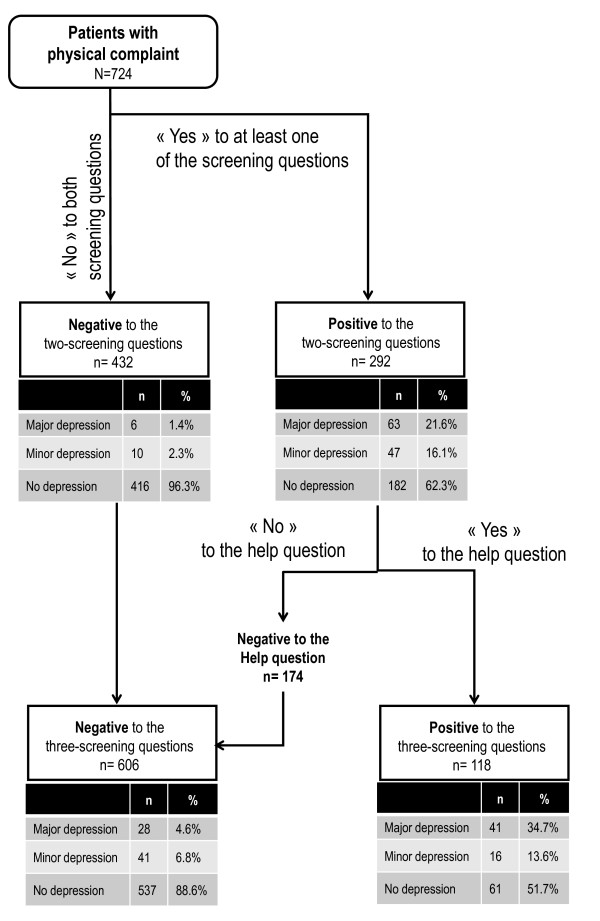
**Flowchart of screening**.

The sensitivity and specificity of the two screening questions were 91.3% (95% CI 81.4 to 96.4) and 65.0% (95% CI 61.2 to 68.6), respectively (Table 1). Adding the 'help' question improved the specificity to 88.2% (95% CI 85.4 to 90.5), but the sensitivity decreased to 59.4% (95% CI 47.0 to 70.9). In fact, 118 (40.4%) of the patients initially screened positive for depression (N 292) were willing to accept help (Figure 2). Considering the patients who were not already being treated for major depression only, the sensitivity and the specificity of the two-question method are, respectively, 84.6% (95% CI 54.6 to 98.1) and 76.8% (95% CI 72.0 to 81.2). For the three-question method the sensitivity decreased to 46.2% (95% CI 19.2 to 74.9) and the specificity increased to 94.5% (95% CI 91.5 to 96.7).

**Table 1:** Sensitivity, specificity, positive/negative predictive values, positive/negative likelihood ratios for major depression

Parameter	Two screening questions % (95% CI)	Three screening questions % (95% CI)
Sensitivity	91.3% (81.4 to 96.4)	59.4% (47.0 to 70.9)
Specificity	65.0% (61.2 to 68.6)	88.2% (85.4 to 90.5)

Positive predictive value	21.6% (17.1 to 26.8)	34.7% (26.4 to 44.1)
Negative predictive value	98.6% (96.8 to 99.4)	95.3% (93.3 to 96.8)

Positive likelihood ratio	2.6 (2.3 to 3.0)	5.0 (3.8 to 6.7)
Negative likelihood ratio	0.1 (0.06 to 0.28)	0.5 (0.3 to 0.6)

We next explored the sensitivity and specificity of both screening instruments in various patient subpopulations (Table 2). The sensitivity of the two-question method was high and consistent through the entire population, ranging from 80% (95% CI 51.3 to 94.6) in patients older than 65 years to 100% (95% CI 83.4 to 100) in men. The specificity of both screening instruments exhibited important disparities across patients with various mental states. Patients who suffered from depression at the index consultation, who were exposed to a psychosocial stressor during the 4 previous weeks, or who were diagnosed with either anxiety or somatoform disorder were more likely to answer positively to each screening instrument without being diagnosed as having depression, as indicated by a lower specificity (Table 2).

**Table 2:** Stratified specificity of screening questions for major depression

Characteristic	Prevalence of depression, % (95% CI)	Specificity, % (95% CI)	
		
		Two screening questions	Three screening questions
Overall	9.5% (7.6 to 12.0)	65.0% (61.2 to 68.6)	88.2% (85.4 to 90.5)
Gender			
Male	9.4% (6.3 to 13.7)	69.2% (63.0 to 75.0)	90.0% (85.4 to 93.4)
Female	9.6% (7.1 to 12.8)	62.5% (57.7 to 67.2)	87.2% (83.5 to 90.2)
Age			
< 65 years	10.9% (8.4 to 14.1)	66.1% (61.4 to 70.5)	87.4% (83.8 to 90.3)
≥ 65 years	6.4% (3.8 to 10.6)	62.8% (56.0 to 69.2)	89.9% (84.9 to 93.4)
Nationality			
Swiss	8.0% (6.0 to 10.6)	67.6% (63.3 to 71.5)	89.1% (86.0 to 91.5)
Not Swiss	14.8% (9.4 to 22.5)	53.2% (43.4 to 62.7)	84.4% (75.9 to 90.4)
Education level			
Professional training	9.4% (71.5 to 12.3)	66.8% (62.4 to 70.9)	88.3% (85.1 to 91.0)
No professional training	9.0% (5.0 to 15.1)	60.6% (51.7 to 68.8)	88.6% (81.7 to 93.3)
Psychosocial stressors			
≥ 1 major stressor	21.5% (16.9 to 26.9)	44.3% (37.6 to 51.1)	77.6% (71.4 to 82.8)
No major stressor	2.0% (1.0 to 4.0)	76.0% (71.7 to 79.9)	93.7% (90.9 to 95.7)
Mood disorders 1 year previously			
Major depression	39.0% (28.6 to 50.4)	34.0% (21.6 to 48.8)	62.0% (47.1 to 75.0)
Minor depression	15.0% (7.5 to 27.1)	43.1% (29.6 to 57.7)	84.3% (70.8 to 92.5)
No depression	39.1% (25.2 to 59.5)	70.1% (66.0 to 73.9)	91.1% (88.3 to 93.3)
Anxiety			
Anxiety syndrome	60.0% (45.2 to 73.2)	5% (0.2 to 26.9)	40.0% (20.0 to 63.6)
No anxiety	57.1% (41.2 to 78.3)	67.1% (63.3 to 70.8)	89.8% (87.1 to 92.0)
Somatoform disorder			
≥ 3 symptoms	31.7% (22.1 to 43.0)	46.4% (33.2 to 60.1)	67.9% (53.9 to 79.4)
< 3 symptoms	6.7% (5.0 to 9.0)	67.7% (62.7 to 70.4)	90.1% (87.4 to 92.3)

## Discussion

In primary care patients well known by their GPs, the two-question screening method for major depression displayed high sensitivity (91%) and low specificity (65%). As suspected, adding the 'help' question led to a decreased sensitivity (59%) but a higher specificity (88%). We also observed a lower specificity for the two-question and three-question methods in subpopulations with other psychiatric conditions (such as generalised anxiety) and in patients who had exhibited major depression 1 year previously.

The strengths of our study are its large sample size, the number and diversity of the participating GPs, and the use of standardised, validated measures for mental disorders. Furthermore, the random selection of patients and their recruitment from a large number of GPs in various settings decreased the risk of selection bias. We therefore believe that our observations are relevant for most patients with physical complaints in primary care in developed countries. However, our study is limited because the two screening questions for major depression were similar to those of the PHQ-9, our reference standard. Therefore, the sensitivity of the screening method is expected to be very high. Finally, the PHQ-9 may not be the best reference standard for major depression for the following three reasons: (1) it is self-report, (2) it doesn't apply exclusion criteria, and (3) it doesn't apply clinical significance criteria. Thus PHQ-9 can only be interpreted as a proxy of DSM-IV [21,22]. Therefore a standardised visit to a psychiatrist would have been preferred.

Whooley *et al. *[23] and Arroll *et al. *[24] first introduced the two-question screening method and reported high sensitivities (96% and 97%, respectively) and low specificities (57% and 67%, respectively). Löwe *et al. *[25] evaluated the two screening questions in outpatients and obtained similar results with a dichotomous answer (yes/no). Furthermore, the two-question method was able to detect changes in a patient's state of depression. Here we report observations similar to those of Arroll *et al*. [24] regarding screening for major depression with two questions. The high sensitivity of these questions allows GPs to securely rule out negative patients, but the relatively low specificity requires further investigations to confidently diagnose major depression in positive cases [14].

Introduction of the third 'help' question was a very interesting and logical proposition, and should have facilitated the diagnosis of major depression. When we added the 'help' question to the screening method, however, our observations were substantially different from those of Arroll *et al*., [15] who reported increased specificity (89%) but identical sensitivity (96%). As an important number of their patients with major depression responded 'no' to the 'help' question, it is not clear why the sensitivity remained identical. In a second study, Goodyear *et al*. [16] validated the two-question and three-question methods using the PHQ-9 as a reference standard for major depression. Although the two-question method was associated with a sensitivity of 98% and a specificity of 73%, and the specificity of the three-question method questions was reported to be 99%, the sensitivity of the three-question method was not provided. A recent publication by the same authors determines a sensitivity of 99.2% and a specificity of 70.4% for the two-question method, whereas the sensitivity decreased to 87.1% and the specificity increased to 94.8% for the three-question method [26].

An independent study by Baker-Glenn *et al*. [27] observed a sensitivity of 23.7% and specificity 97.8% in patients attending chemotherapy with the three-question method. We therefore believe Arrol *et al*.'s [15] results to be misleading. These findings support the latest NICE [28] guidelines that recommend only the use of the two screening questions.

Our analysis indicates that although the three-question method has high negative predictive value, the high false negative rate implies that as many as four patients out of ten (28/69) with major depression would not be correctly diagnosed with this method. In comparison, less than one out of ten patients (6/69) with major depression will not be diagnosed when using the two-question method. It is therefore not helpful to include the third 'help' question to rule out major depression in patients well known by their GPs. But as Kroenke [29] suggests, 'screening for depression is not enough'. Patients identified with depression have to be treated. Therefore the 'help' question remains clinically relevant, even if more than half of patients with major depression did not ask for help. But within the context of the consultation, the 'help' question enables a continuing discussion about mood disorders and allows evaluation of the appropriateness of a psychiatric treatment and referral. Baker-Glenn *et al*. conclude, as we do, that the 'help' question may highlight patients willing to accept support [27]. This also underlines GPs' role in investigating and answering patient expectations for their psychological distress as described by Walters showing that patients with milder symptoms usually prefer simple human contact, and informal resource rather than formal interventions or medication [30]. While all these questions may help GPs screen for major depression in their patients, this tool should not replace clinical judgment; indeed, GPs seldom rely on questionnaires alone [31,32].

Our observations suggest that the sensitivity of the two screening questions is consistent across various patient subpopulations guaranteeing a low number of false negatives regardless of patient characteristics. However, as the specificity differs across patients, GPs may frequently and falsely diagnose major depression in patients who present other mental disorders. Additional studies are necessary to quantify the actual benefits of screening mental disorders in primary care with the two-question and three-question screening methods.

## Conclusions

The two-question screening method for major depression exhibited a high sensitivity and a low specificity when applied to well known primary care patients with a physical complaint. Adding the 'help' question improved the specificity of the test, but clearly decreased its sensitivity: four out of ten patients will thus be missed with the three-question method, compared to only one out of ten with the two-question method. Although the 'help' question is not useful as a screening question in this patient group, it may facilitate discussion about mood disorders and its management.

## Competing interests

All authors had full access to all data (including statistical reports and tables) and take responsibility for the integrity of the data and the accuracy of the data analysis. All authors declare that they have no competing interests.

## Authors' contributions

LH, BF, FV, and BB participated in the conception and design of the study. NH, LH, FV, and TB collected data. NH monitored data collection and completed missing data, PL, PV, LH, and BF planned and analysed the data, PL, PV, BF, and LH participated in data interpretation, drafting, and revising the manuscript. NH, BB, BF, FV, and TB reviewed the manuscript. LH is the guarantor of the paper. All authors read and approved the final manuscript.

## Pre-publication history

The pre-publication history for this paper can be accessed here:

http://www.biomedcentral.com/1741-7015/9/114/prepub
